# The coiled-coil domain of zebrafish TRPM7 regulates Mg·nucleotide sensitivity

**DOI:** 10.1038/srep33459

**Published:** 2016-09-15

**Authors:** Chad Jansen, Jaya Sahni, Sayuri Suzuki, F. David Horgen, Reinhold Penner, Andrea Fleig

**Affiliations:** 1 Center for Biomedical Research, The Queen’s Medical Center and University of Hawaii, Honolulu, HI-96813, USA; 2 University of Hawaii Cancer Center and John A. Burns School of Medicine, University of Hawaii, Honolulu, HI-96813, USA; 3 Center for Immunity and Immunotherapies, Seattle Children’s Research Institute, 1900 9th Ave, Seattle, WA 98101, USA; 4 Laboratory of Marine Biological Chemistry, Department of Natural Sciences, Hawaii Pacific University, Kaneohe, HI 96744, USA

## Abstract

TRPM7 is a member of the Transient-Receptor-Potential Melastatin ion channel family. TRPM7 is a unique fusion protein of an ion channel and an α-kinase. Although mammalian TRPM7 is well characterized biophysically and its pivotal role in cancer, ischemia and cardiovascular disease is becoming increasingly evident, the study of TRPM7 in mouse models has been hampered by embryonic lethality of transgenic ablations. In zebrafish, functional loss of TRPM7 (drTRPM7) manifests itself in an array of non-lethal physiological malfunctions. Here, we investigate the regulation of wild type drTRPM7 and multiple C-terminal truncation mutants. We find that the biophysical properties of drTRPM7 are very similar to mammalian TRPM7. However, pharmacological profiling reveals that drTRPM7 is facilitated rather than inhibited by 2-APB, and that the TRPM7 inhibitor waixenicin A has no effect. This is reminiscent of the pharmacological profile of human TRPM6, the sister channel kinase of TRPM7. Furthermore, using truncation mutations, we show that the coiled-coil domain of drTRPM7 is involved in the channel’s regulation by magnesium (Mg) and Mg·adenosine triphosphate (Mg·ATP). We propose that drTRPM7 has two protein domains that regulate inhibition by intracellular magnesium and nucleotides, and one domain that is concerned with sensing magnesium only.

TRPM7 is one of the eight members of the transient receptor potential melastatin (TRPM) cation channel family (reviewed in ref. 1). TRPM7 is a ubiquitously expressed fusion protein that consists of an ion channel linked to an alpha kinase, and therefore the protein is known as a “channel kinase”. It is negatively regulated by intracellular magnesium (Mg^2+^), adenosine triphosphate (Mg·ATP) and other polyvalent molecules^1^.

TRPM7 exhibits an outwardly rectifying current-voltage relationship due to voltage-dependent block by Mg^2+^ and calcium (Ca^2+^) at negative voltages^2^,^3^. A subunit of TRPM7 consists of six trans-membrane spans with a pore loop segment between span five and six that forms the pore and contains the ion selectivity filter^4^. Mouse TRPM7 (mTRPM7) has been shown to best permeate zinc (Zn^2+^) and nickel (Ni^2+^), followed by barium (Ba^2+^), cobalt (Co^2+^), magnesium (Mg^2+^), manganese (Mn^2+^), strontium (Sr^2+^), cadmium (Cd^2+^) and calcium (Ca^2+^)^5^. Based on this profile and the effects of divalent substitution/complementation experiments, the TRPM7 channel function is thought to serve as a primary mechanism for cellular Mg^2+^ homeostasis and be essential for cell proliferation^6^. Disrupting mTRPM7 function either by knock-out of the complete protein or its kinase domain is embryonically lethal^7^,^8^. TRPM7 has been implicated in many diseases, primarily cancer and ischemia^1^, and influx of Zn^2+^ due to TRPM7 activity has been linked to neurotoxicity after brain ischemia^9^. Compromised TRPM7 function has also been proposed to underlie macrothrombocytopenia in humans, partially caused by altered cellular Mg^2+^ homeostasis^10^.

Mouse TRPM7 changes its selectivity profile towards preferring monovalent ions to divalent ions in response to acidic pH^4^, and hyperosmotic conditions inhibit current activity^11^. Several compounds are known to inhibit mammalian TRPM7, including the relatively non-specific compound 2-aminoethyl diphenylborinate (2-APB) and the highly specific blocker waixencin A^12^,^13^. These inhibitors have proven useful in the distinction between human TRPM7 (hTRPM7) and its sister channel, human TRPM6 (hTRPM6), which share ~50% amino acid sequence identity^14^. While 2-APB inhibits hTRPM7 currents, it enhances hTRPM6, and waixenicin A has no significant effect on hTRPM6^14^.

TRPM6 is the sixth member of the TRPM family. Similarly to TRPM7, TRPM6 has both a channel and alpha-kinase domain^15^. In analogy to TRPM7, a subunit of TRPM6 consists of six trans-membrane spans with a pore loop segment between span five and six that forms the pore and contains the ion selectivity filter^15^. The divalent permeability of TRPM6 is similar to TRPM7 with the major difference that Ni^2+^ is less permeable^12^. TRPM6 subunits can form homomeric tetramers as well as heteromeric TRPM7/TRPM6 complexes that have their own unique properties^12^,^14^,^16^. One of the major differences between TRPM7 and TRPM6 is the rather tissue-specific expression of TRPM6, largely restricted to colon, kidney and testis.

Knock-out or disruption of TRPM7 function in mice is embryonically lethal^1^. Zebrafish, on the other hand, survives a loss of function of this protein, which makes it a desirable animal model to study cellular and developmental effects linked to non-functional TRPM7. Forward genetics showed that zebrafish TRPM7 (drTRPM7) is involved in melanophore development, early motility and adult growth^17^,^18^,^19^,^20^. Systemic calcium and magnesium homeostasis is abnormal in drTRPM7 mutants^18^,^21^, and drTRPM7 mutant larvae develop bradycardia^17^. A recent study demonstrated involvement of drTRPM7 in both the function and maintenance of dopaminergic neurons^19^ and wild-type as well as three *touchdown* drTRPM7 mutants have been probed for current activity using two-electrode voltage clamp in *Xenopus* oocytes^20^. Nevertheless, the biophysical properties of drTRPM7 remain unknown. Amino acid sequence analysis shows that drTRPM7 shares 72% sequence identity with hTRPM7, and 49% sequence identity with hTRPM6 (www.uniprot.org). Therefore, it seems plausible to suspect drTRPM7 ion channel behavior might be different from that observed in other species. Understanding drTRPM7 characteristics may provide valuable information when using zebrafish as a disease model for the study of the TRPM7 channel kinase^22^.

## Materials and Methods

### Molecular Biology

#### Cloning and expression analysis of WT and mutated versions of zebrafish TRPM7 (drTRPM7)

Wild type (WT) zebrafish TRPM7 (NP_001025232) was a kind gift from Dr. Michael Elizondo. WT drTRPM7 was restriction digested NotI/NheI and cloned into pcDNA5/TO (Invitrogen) with a hemagglutinin (HA) epitope tag at the N-terminus. All the subsequent mutant versions were cloned into HA-tagged pcDNA5/TO (Invitrogen) for tetracycline/doxycycline-regulated expression using either NotI/SpeI or NotI/KpnI restriction sites. Post cloning, sequencing was carried out to ensure that the coding sequence was in-frame for each construct.

To amplify cDNA for transfection of tetracycline-inducible HEK293 T-REx cells, competent DH5 alpha cells (Invitrogen Cat. No. 18258-012) were transformed with a pcDNA5/TO plasmid (Invitrogen Cat. No. V1033-20) harboring either drTRPM7 wild type (drTRPM7 WT), drTRPM7-1478, drTRPM7-1258 or drTRPM7-1178 truncations.

#### Gene overexpression system

Stable, inducible overexpression HEK293 T-REx cell lines wild type and mutant drTRPM7 were generated according to manufacturer’s instructions (Invitrogen, USA). Immunoblot analysis was performed to verify expression of drTRPM7 wild type or mutants.

### Immunoblot analysis

For immunoblot analysis, HEK293 T-REx drTPRM7 wild type or truncation mutant cells were induced for 16 h with 1 μg/mL tetracycline added to the media. Cells were harvested and dissolved in lysis buffer (10 mM Tris-HCl, 75 mM NaCl, 5% glycerol, 0.5% triton, 5 mM EDTA, 1 mM PMSF) containing protease inhibitors (104 mM AEBSF, 80 μM Aprotinin, 4 mM Bestatin, 1.4 mM E-64, 2 mM Leupeptin and 1.5 mM Pepstatin A) for 30 minutes at 4 °C. After incubation, the lysates were centrifuged at 12,000 rpm for 5 min. at 4 °C and the protein concentration of the lysates were measured using a protein assay reagent (Thermo Scientific, USA). Soluble lysates were boiled with NuPAGE LDS sample buffer and NuPAGE Reducing Agent (Invitrogen) at 95 °C for 8 minutes. Equal amounts of proteins (40 μg) were loaded and separated in a NuPAGE 4–12% gel (Invitrogen) then transferred to a PVDF membrane. Proteins were detected by immunoblotting with the antibodies anti-HA (3F10, Roche), anti-GAPDH (6C5, Abcam, UK) followed by treatment with horseradish peroxidase (HRP)-conjugated anti-rat or anti-mouse antibody (GE Healthcare, USA). GAPDH was evaluated as an internal control. The antibody-bound protein was visualized using ECL solution (Life Technologies, USA.)

### Cell Culture

HEK293 T-REx cells stably expressing HA-tagged *Danio rerio* TRPM7 (drTRPM7) wild type or mutants were cultured in DMEM with 10% FBS and 5 μg/ml of blasticidin and 200 μg/ml hygromycin. HEK293 T-REx cells were incubated at 37 °C and 5% CO_2_. Expression was induced by adding 1 μg/ml tetracycline to the growth media 20 hours before experiments. The adult liver zebrafish cell line (ZFL; ATCC Cat. No. CRL-2643) was kept in the media suggested by ATCC (50% L-15 (ATCC 30-2008) 35% DMEM HG (GIBCO, USA) 15% Ham’s F12 (GIBCO) supplemented with 0.15 g/L sodium bicarbonate 15 mM HEPES, 0.01 mg/ml bovine insulin, 50 ng/ml mouse EGF, 5% heat-inactivated fetal bovine serum 0.5% Trout Serum) at 28 °C with no extra CO_2_. Chicken DT40 wild type and DT40 cells with a TRPM7 knock-down (DT40 TRPM7-KO) cells were maintained as described before^6^.

### Electrophysiology

Patch-clamp experiments were performed in the tight-seal whole-cell configuration at room temperature (20–25 °C). Whole-cell currents were recorded with the EPC9 (HEKA, Bellmore, NY) and Patchmaster v2.4 (HEKA). Voltages were corrected for a liquid junction potential of 10 mV. The patch pipettes were pulled from borosilicate glass (Sutter Instruments, USA) with resistances of 2.5–3.5 MΩ when filled with internal solution. The voltage ramp protocol ranged from −100 mV to 100 mV over 50 ms and applied at 0.5 Hz. Current amplitudes were measured at −80 mV and +80 mV. The holding potential was set to 0 mV in all experiments. Currents were filtered at 2.9 kHz and digitized at 10 kHz. Cells were seeded on glass cover slips 1–2 days before the day of the experiment. Cover slips were submerged in the external solution at room temperature (20–25 °C). Cover slips and external solutions were replaced every hour.

### Solutions

The standard external solution contained (in mM): 140 NaCl, 2.8 KCl, 2 MgCl_2_, 11 glucose, 10 HEPES, and 1 CaCl_2_. The divalent profile experiments were performed using a modified external solution that contained (in mM): 140 N-methyl-D-glucamine (NMDG), 11 glucose, 10 HEPES and 10 CaCl_2_. For application, 10 mM CaCl_2_ was replaced with 10 mM of the respective divalent under study. The standard internal solution contained (in mM): 140 Cs-glutamate, 8 NaCl, 10 HEPES, and 10 Cs-BAPTA. pH was adjusted to 7.2 and osmolarity was kept between 290–330 mOsm. The solutions for pH below 6.0 contained (in mM): 140 NaCl, 2.8 KCl, 2 MgCl_2_, 11 glucose, 10 MES, and 1 CaCl_2_. For the Mg·ATP dose-response analysis, the internal solution contained (in mM): 120 Cs-glutamate, 8 NaCl, 10 HEPES, 10 Cs-BAPTA. Na·ATP and MgCl_2_ was used to achieve appropriate concentrations of free intracellular Mg^2+^ and Mg·ATP as calculated using WebmaxC Standard (http://www.stanford.edu/~cpatton/webmaxcS.htm). Waixenicin A was isolated and purified to >95% purity from the soft coral *Sarcothelia edmonds*oni as described previously^13^, and single use aliquots were reconstituted in methanol and diluted to 10 μM in standard external solution.

### Analysis

Statistical analysis was performed using Fitmaster (version 2.69; HEKA, Germany) and Igor software (version 6.34; WaveMetrics, USA). The p-values were calculated using Microsoft Excel version 14.1.2 using the two-tailed paired Student’s T-test. Data were normalized to cell size and expressed as current density (pA/pF), or normalized to the time point right before application (I/I_[time]s_). Data were displayed as mean ± standard error of mean (s.e.m.). Dose-response fits were assessed using the fit function f(x) = ([Maximum current] *(1/(1 + ([Estimated IC_50_]/x)^[Hill coefficient])). The IC_50_ or EC_50_ values calculated with the dose-response curve function were reported with ± standard deviation (S.D.).

### Animals

No live vertebrate animals were used in this study.

## Results

### Mg^2+^ and Mg·ATP sensitivity of zebrafish TRPM7 is similar, but not identical to mammalian TRPM7

**Figure 1:**
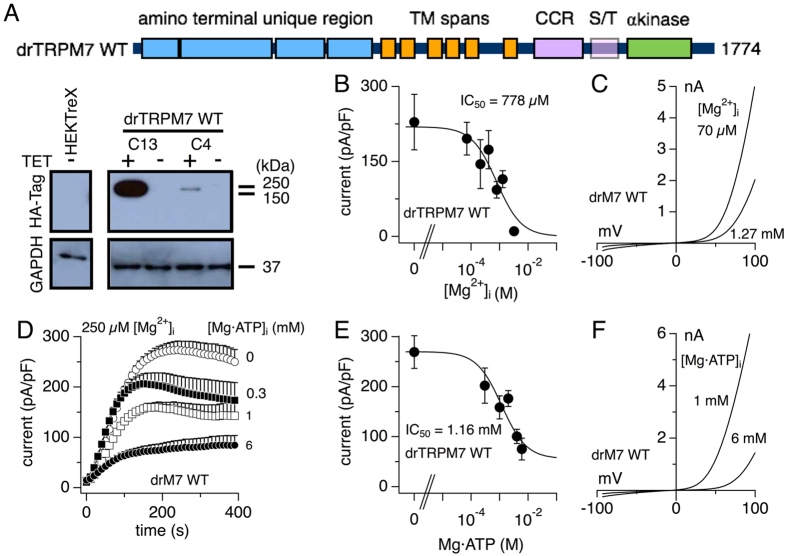
Mg^2+^ and Mg·ATP sensitivity in drTRPM7. (**A**) Schematic of wild type zebrafish TRPM7 (drM7 WT) and anti-HA immunoblot analysis of HEK293 T-REx cells with or without tetracycline-regulated expression of zebrafish HA-TRPM7 wild type having survived the selection process. Clone 13 and clone 4 are depicted, either exposed to tetracycline (+) or not (−). TM = transmembrane; CCR = coiled-coil region; S/T = Serine/threonine rich region; TET = tetracycline. (**B**) To assess the dose-response of drTRPM7 to increasing intracellular free Mg^2+^, current amplitudes were extracted at +80 mV and 200 s, normalized to cell size, averaged and plotted against the respective Mg^2+^ concentration. A dose response fit yielded an IC_50_ of 778 ± 291 μM with the Hill coefficient kept at 1. Error bars indicate s.e.m. (**C**) Representative current-voltage (I/V) curves of drTRPM7 at 70 μM and 1.27 mM free intracellular Mg^2+^. (**D**) Whole cell currents in HEK293 T-REx cells over-expressing drTRPM7 were measured with an internal solution containing 250 μM free Mg^2+^ and varying levels of Mg·ATP (n = 6–12). Current amplitudes were extracted at +80 mV, normalized to cell size, averaged and plotted against time. (**E**) To assess the dose-response of drTRPM7 to intracellular Mg·ATP, current amplitudes of the data in (**D**) were extracted at 200 s and plotted against the respective Mg·ATP concentration. A dose-response fit yielded an IC_50_ 1.16 ± 0.7 μM with the Hill coefficient set to 1. (**F**) Representative I/V curves of drTRPM7 at 1 mM and 6 mM intracellular Mg·ATP.

To study channel biophysics, HA-tagged wild type zebrafish TRPM7 (drTRPM7) was cloned and stably expressed in HEK293 T-REx cells under tetracycline-inducible promotor (see methods; ref. 3). Thirteen drTRPM7-HEK293 clones were established, out of which clone 4 and clone 13 showed a signal at the correct size when probed for the HA-tag via Western Blotting (Fig. 1A). Both clones were also tested for drTRPM7 expression using the whole-cell patch-clamp technique. Clone 4 was chosen for further analysis, as clone 13 tended to lose drTRPM7 expression after only few passages in cell culture despite continued selection pressure by blasticidin and hygromycin. To assess whether the biophysical characteristics of drTRPM7 were similar to those of mammalian TRPM7, we performed a dose-response analysis of whole-cell currents under various concentrations of internal free Mg^2+^ in HEK293 T-REx cells overexpressing wild type drTRPM7. A dose-response curve fit to averaged currents extracted at 200 s into the experiment revealed a half-maximal inhibitory concentration (IC_50_) of 778 ± 291 μM with a Hill coefficient of 1 (Fig. 1B). The current-voltage relationship exhibited the typical TRPM7 signature with large outward and small inward currents that were both suppressed under higher internal Mg^2+^ concentrations (Fig. 1C).

A dose-response analysis of drTRPM7 whole-cell currents to increasing internal Mg·ATP concentrations while clamping internal Mg^2+^ to 250 μM revealed an IC_50_ of 1.16 ± 0.7 mM with a Hill coefficient of 1 (Fig. 1D,E). Figure 1D shows drTRPM7 current development over time at increasing concentrations of internal Mg·ATP and normalized to cells size. Notably, 6 mM Mg·ATP was not sufficient to completely suppress drTRPM7 and about 30% of current activity remained (Fig. 1E,F). We conclude that the sensitivity of drTRPM7 to intracellular Mg^2+^ and Mg·ATP is similar to what is observed for hTRPM7 (Table 1). However, a residual current remains, which resembles the inability of Mg·ATP to suppress TRPM6 channel activity^14^.

### Divalent conductance profile of drTRPM7

**Figure 2:**
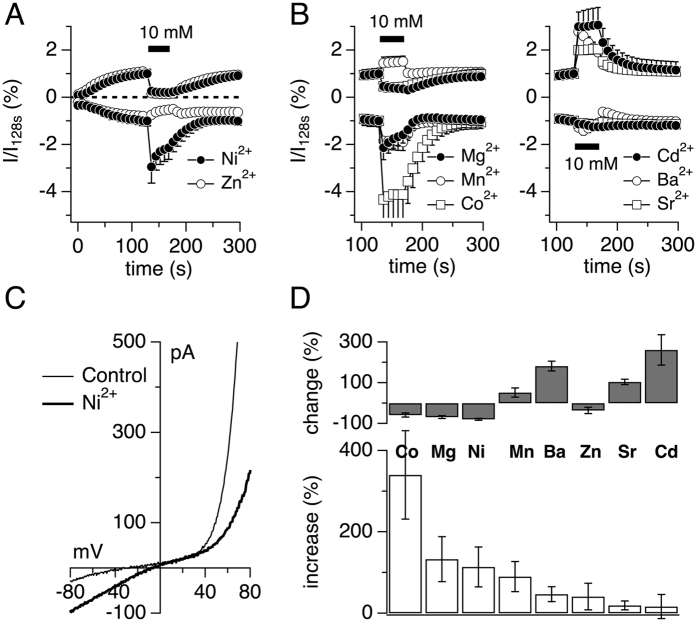
Divalent profile of drTRPM7. (**A,B**) Whole cell currents in HEK293 T-REx cells over-expressing drTRPM7 were measured with an external solution containing 10 mM Ca^2+^. At 128 s into the experiment, 10 mM Ca^2+^ was replaced with the respective equimolar divalent as indicated. Currents were measured at +80 mV, normalized to 128 s, averaged and plotted against time (n = 4–6). (**C**) Representative I/V curve of drTRPM7 exposed to 10 mM Ca^2+^ (128 s; thin line) or 10 mM Ni^2+^ (150 s; thick line). (**D**) The bar graph depicts the change (%) in drTRPM7 outward currents and increase (%) in drTRPM7 inward currents (lower panel) at peak current amplitudes during the respective external divalent ion exchange. Error bars indicate s.e.m.

The TRPM7 channel domain is unique in that it allows conductance of a range of divalent ions^3^,^5^. To confirm this characteristic in drTRPM7, experiments were performed as previously described^5^. Briefly, HEK293 T-REx overexpressing drTRPM7 were exposed to the standard external solution supplemented with 10 mM CaCl_2_. Whole-cell currents were allowed to develop fully and replaced by a solution containing an equimolar concentration of a divalent cation of interest applied for 40 s at 130 s into the experiment in a solution with NMDG replacing Na^+^ (Fig. 2A,B). Peak inward currents during application were extracted at −80 and +80 mV and plotted according to the amount of current increase assessed at −80 mV (Fig. 2D). This revealed that TRPM7 conducted Ca^2+^ the least compared to other divalents, with cobalt and magnesium permeating the best. In addition, monovalent outward currents were differentially affected by extracellular divalent application. While cadmium, strontium and barium facilitated outward currents (Fig. 2B,D), nickel, magnesium and cobalt almost completely suppressed monovalent ion flux through TRPM7 (Fig. 2A,D). The current-voltage behavior before and during nickel exposure is exemplified in Fig. 2C. Note the right-shift of the reversal potential under Ni^2+^ exposure.

### pH and sensitivity to osmolarity in drTRPM7

**Figure 3:**
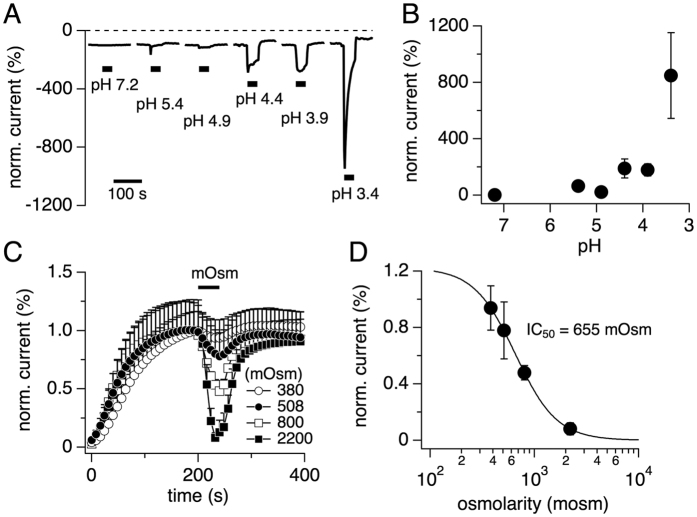
Effect of pH and osmolarity on drTRPM7. (**A**) Effect of extracellular acidification on whole-cell currents in HEK293 T-REx cells over-expressing drTRPM7. Currents were evoked by the standard voltage ramp from −100 mV to +100 mV and analyzed at −80 mV to assess the development of inward current amplitudes over time. Currents were normalized to 200 s, just before external solution exchange by application. Black bars indicate application of external solutions with decreasing pH values. Each data trace represents an individual sample cell. HEPES buffer was used for solutions with a pH > 6 and MES buffer was used for solutions pH < 6. (**B**) Normalized average peak inward currents measured during pH applications and plotted against their respective pH value (n = 4–15). Data acquisition as described in panel (**A**). Error bars indicate s.e.m. (**C**) Normalized and averaged whole cell currents in HEK293 T-REx cells over-expressing drTRPM7 and application of external solutions that increased in osmolarity (n = 4–10). Current amplitudes were analyzed at +80 mV. Black bar indicates time of application. (**D**) Normalized average peak current inhibition of drTRPM7 induced by increasing osmotic pressure. Data taken from panel (**C**). The dose-response fit yielded an IC_50_ of 655 ± 7.3 mOsm (Hill = 2.18).

TRPM7 currents are sensitive to acidic pH conditions and are inhibited by high osmotic pressure^4^,^11^. To assess pH sensitivity, HEK293 T-REx overexpressing drTRPM7 were probed with the standard voltage ramp protocol using the whole-cell patch-clamp technique (see methods), and external solutions adjusted to different pH values were applied for 40 s as indicated by the black bars in Fig. 3A. Analyzing the peak inward current during the pH change and plotting the averaged values against their respective pH revealed the all-or-none behavior of drTRPM7 at different external pH, greatly enhancing TRPM7 currents between pH 3.9 and pH 3.4 (Fig. 3C). To assess drTRPM7 sensitivity to osmotic changes, heterologously expressing drTRPM7 cells were exposed for 40 s to external solutions with different osmotic values (Fig. 3B). Maximal current inhibition during the application period was assessed at +80 mV and plotted against the respective osmolarity value (Fig. 3D). The resulting dose-response curve revealed that hyperosmotic conditions inhibited drTRPM7 with an IC_50_ of 655 ± 8.3 mOsm and a Hill coefficient of 2.2 (Fig. 3D). We conclude that drTRPM7 and hTRPM7 have similar characteristics in regards to pH and osmolarity^11^.

### Pharmacological profile of drTRPM7

**Figure 4:**
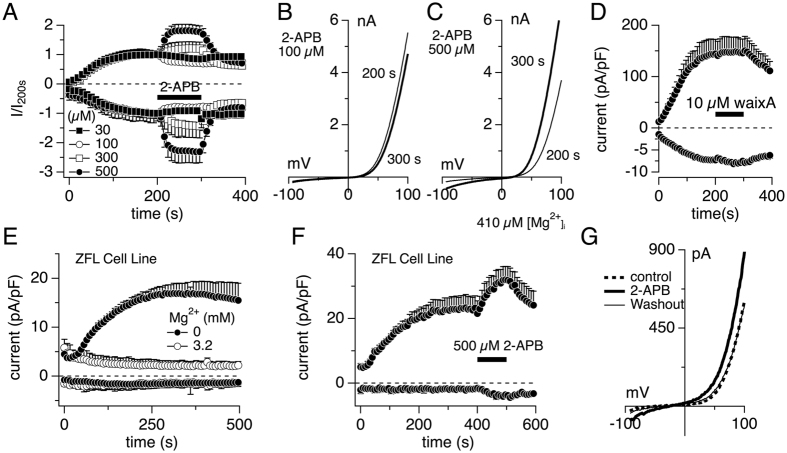
Heterologous and endogenous drTRPM7 is facilitated by 2-APB. (**A**) Application of 2-APB on whole-cell currents in HEK293 T-REx cells overexpressing drTRPM7 as indicated by the black bar (n = 5–6). Currents were analyzed at −80 mV and +80 mV, normalized to the current at 200 s and plotted over time of the experiment. Error bars indicate s.e.m. (**B**) Representative I/V curve of drTRPM7 before (thin line) and during (thick line) 100 μM 2-APB application. (**C**) Representative I/V curve of drTRPM7 currents before (thin line) and during 500 μM 2-APB application (thick line). (**D**) Development of whole-cell currents in HEK293 T-REx cells overexpressing drTRPM7 and analyzed at −80 mV and +80 mV. The black bar indicates application of 10 μM waixenicin A (n = 6). Currents were analyzed as in (**A**) and normalized to cell size. Intracellular solution contained 780 μM free Mg^2+^. (**E**) Endogenous TRPM7-like whole-cell currents assessed in the zebrafish liver (ZFL) cell line. Currents were analyzed at −80 mV and +80 mV, normalized to cell size, averaged and plotted versus time. The internal solution contained either EDTA and no Mg^2+^ or 3.2 mM free Mg^2+^ (n = 4 and n = 5, respectively). (**F**) Current development of drTRPM7-like whole-cell currents measured in ZFL cells and analyzed as in (**E**). Cells were superfused with 500 μM of 2-APB between 400 s and 500 s as indicated by the black bar (n = 4). (**G**) Representative I/V curve of endogenous drTRPM7-like current before, during and after application of 500 μM 2-APB.

hTRPM7, but not human TRPM6 (hTRPM6), is inhibited by the non-selective compound 2-aminoethyl diphenylborinate (2-APB)^12^,^14^, and the potent and selective inhibitor waixenicin A^13^. Testing various concentrations of 2-APB in HEK293 T-REx cells overexpressing drTRPM7 showed that this compound facilitated rather than inhibited drTRPM7 currents, even at concentrations as high as 500 μM (Fig. 4A). This is illustrated by current-voltage curves recorded before (200 s) and during (300 s) 2-APB application, clearly demonstrating increased inward and outward drTRPM7 currents during compound application (Fig. 4B,C). Furthermore, application of 10 μM waixenicin A had no effect on drTRPM7, despite the presence of 410 μM internal free Mg in the pipette solution (Fig. 4D). We conclude that the pharmacological profile of drTRPM7 with respect to both 2-APB and waixenicin A is more similar to that seen in hTRPM6 rather than hTRPM7.

### Native TRPM7-like currents in a zebrafish liver cell line are facilitated by 2-APB

As in mammalian tissues, TRPM7 appears to be ubiquitously expressed in zebrafish as determined by *in situ* hybridization^20^. The protein most similar to TRPM7 is TRPM6. In zebrafish, TRPM6 expression levels vary with tissue as determined by RT-qPCR: While highly expressed in gut, gills and operculum, liver tissue shows negligible drTRPM6 expression^23^. To confirm that the pharmacological profile of overexpressed wild type drTRPM7 can be replicated in the native system, we performed whole-cell patch-clamp experiments using the zebrafish liver cell line ZFL (ATCC Number CRL-2643). Endogenous TRPM7-like currents were allowed to develop in the absence of intracellular Mg^2+^ by supplementing the internal solution with 10 mM Cs-EDTA (Fig. 4E). This revealed a small outwardly rectifying current with the signature current-voltage relationship of overexpressed drTRPM7 (Fig. 4G). Addition of 3.2 mM free Mg^2+^ to the intracellular pipette solution completely suppressed drTRPM7-like current development (Fig. 4E), while application of 500 μM 2-APB at 400 s into the experiment strongly facilitated both inward and outward currents by 50% (Fig. 4F,G). We conclude that endogenous TRPM7-like currents in the ZFL cell line mimic the 2-APB profile of heterologously expressed drTRPM7.

### C-terminal truncations of drTRPM7 rescue cell growth in TRPM7-deficient DT40 B cells

**Figure 5:**
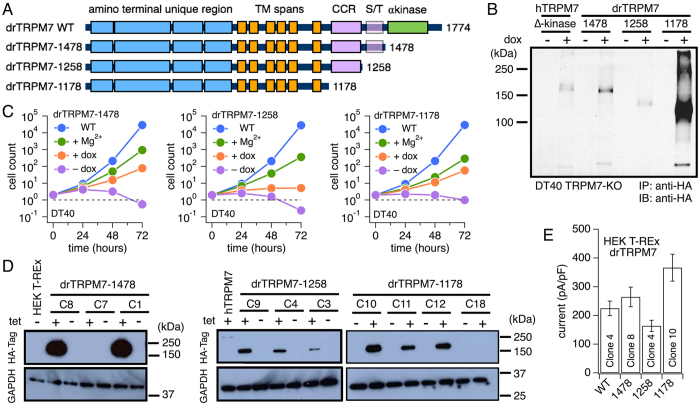
DT40 TRPM7-KO cells and HEK293 T-REx cells overexpressing drTRPM7 truncation mutants. (**A**) Schematic of drTRPM7 and location of truncations. Abbreviations as in Fig. 1. (**B**) Immunoblot of human ∆-kinase and truncated mutants of drTRPM7 in chicken DT40 B cells deficient in endogenous TRPM7 (DT40 TRPM7-KO)^6^. DT40 TRPM7-deficient cells overexpressing either human TRPM7 ∆-kinase or three zebrafish TRPM7 truncation mutants were induced with doxycycline for 48–72 hours (+dox) and harvested. Their expression was analyzed by SDS-PAGE and immunoprecipitation (IP) with an anti-HA antibody followed by immunoblotting (IB) with anti-HA. Doxycycline non-induced cells (-dox) were used as a negative control. (**C**) Growth curves of truncated mutants of drTRPM7 in DT40 TRPM7-KO cells. DT40 TRPM7-KO cells expressing different truncated versions of drTRPM7 were induced with doxycycline (dox) in RPMI without 15 mM supplemental Mg^2+^ and their growth was assessed up to 72 hours. DT40 wild type cells (DT40 WT) and untransfected DT40 TRPM7-KO cells with 15 mM supplemental Mg^2+^ were used as positive controls. DT40 TRPM7-deficient cells expressing the mutant(s) but kept uninduced and without 15 mM supplemental Mg^2+^ were used as a negative control. Cell growth was plotted on an exponential scale. WT = wild-type; +Mg^2+^ = TRPM7-KO cells with 15 mM supplemental Mg^2+^ added to the growth medium; -dox = no doxycycline added; +dox = 1 μg/ml doxycycline added. (**D**) Anti-HA immunoblot analysis of HEK293 T-REx cells with tetracycline-regulated (tet) expression of zebrafish HA-TRPM7 truncation clones [C] having survived the selection process. (**E**) Peak average current densities (pA/pF) of heterologous drTRPM7 wild type and truncation mutants in the plasma membrane of tet-induced HEK293 T-Rex at 20 hours of induction and intracellular solution void of Mg^2+^ and supplemented with 1 mM EDTA (n = 5–6; error bar = s.e.m.).

Several findings in human and mouse TRPM7 C-terminus truncation and point mutants alter the channel’s constitutive activity and sensitivity to Mg^2+^ and Mg·ATP^1^. For example, truncating the mTRPM7 kinase domain at aa 1599 renders a non-functional channel^24^, while cutting mTRPM7 at aa 1510 regains regular-sized currents with a current-voltage behavior typical for TRPM7^25^. Point mutations in the hTRPM7 kinase domain targeting the Mg·ATP binding site desensitizes the channel to Mg^2+^ and Mg·NTPs, whereas deleting the kinase domain at aa 1569 significantly enhances channel sensitivity to these agents^6^. We hypothesized that the region between the channel domain and the kinase domain might be involved in coordinating inhibitory Mg^2+^ and Mg·nucleotide regulation, and that one of the two postulated Mg^2+^ regulatory sites would reside within that region^6^. To test whether this scenario applies to drTRPM7 as well, we constructed three drTRPM7 truncation mutants by cutting the C-terminus at aa positions 1478, 1258 and 1178 (see methods). The drTRPM7 mutant cut at position 1478 (drTRPM7-1478) effectively removes the protein’s α-kinase domain, leaving the serine-threonine-proline-rich region (S/T-rich) and coiled-coil (CC) region intact (Fig. 5A). The drTRPM7-1258 mutant retains the CC domain, and the drTRPM7-1178 mutant is cut just after the six trans-membrane domains forming the ion channel pore, leaving just the TRP box intact.

Doxycycline-inducible chicken DT40 B cells overexpressing HA-tagged drTRPM7 truncation mutants were established in a TRPM7-deficient background (DT40 TRPM7-KO)^6^ and tested for heterologous protein expression by Western blot (Fig. 5B). It has been shown previously that DT40 cell growth is dependent on TRPM7 expression, and TRPM7-deficiency can be rescued by either high extracellular Mg^2+^ supplementation of the growth media or overexpression of alternate Mg^2+^ transporters, including TRPM7 from species other than chicken^6^. When comparing growth curves of DT40 B cells overexpressing drTRPM7 truncation mutants, it can be seen that all three mutants were able to restore cell growth compared to TRPM7-deficient cells (Fig. 5C). While drTRPM7-1478 and drTRPM7-1178 mutants were similarly effective in growth rescue and drTRPM7-1258 appeared somewhat less effective, all three mutants were able to rescue cell growth compared to control. We conclude that the three drTRPM7 truncation mutants are able to maintain channel activity.

To analyze the biophysical behavior, drTRPM7 truncation mutants were subsequently cloned into the standard tetracycline-inducible HEK T-Rex cell system^3^ (Fig. 5D). Three clones survived the selection process for TRPM7-1478 and TRPM7-1258, and four clones survived for TRPM7-1178. All clones were tested for expression levels by immunoblotting (Fig. 5D). Since Western blot reflects global cellular protein expression, we assessed all positive clones for plasma membrane expression using the whole-cell patch clamp technique. To this end, the internal solution was kept Mg^2+^- and Mg·ATP-free and supplemented with 1 mM EDTA to maximally activate drTRPM7 currents. Clones, where each tested cell consistently ad currents in the nano Ampere (nA) range were chosen for further analysis (Fig. 5E).

### C-terminal truncation of drTRPM7 at amino acid 1178 restores normal Mg^2+^ sensitivity

**Figure 6:**
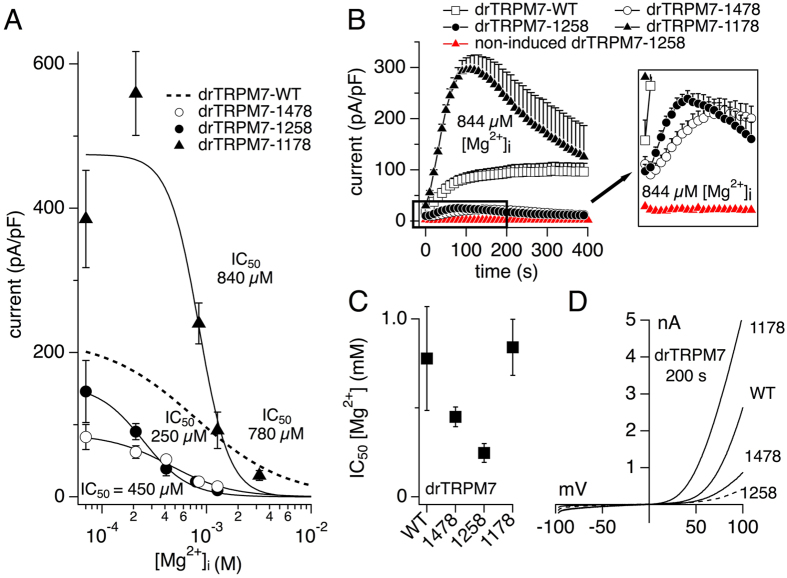
Truncation of drTRPM7 at amino acid 1178 retains inhibitory efficacy of internal Mg^2+^. (**A**) drTRPM7 truncation mutants overexpressed in tetracycline-induced HEK293 T-REx cells were subjected to a Mg^2+^ dose-response curve analysis using the whole-cell patch clamp technique. drTRPM7 currents were allowed to develop over time, and current amplitudes were measured at +80 mV, extracted at 200 s, normalized for cell size, averaged and plotted versus the respective internal Mg^2+^ concentration (n = 4 to 10). Error bars indicate s.e.m. Each respective concentration-response curve was fitted with a dose-response fit to obtain their IC_50_. Dotted line represents the dose-response fit to wild type drTRPM7 as depicted in Fig. 1. (**B**) Whole-cell current development of tetracycline-induced wild type and drTRPM7-truncation mutants in the presence of 844 μM free intracellular Mg^2+^ (black symbols; n = 4 to 10; left panel). In red is the current development of endogenous hTRPM7 under identical conditions in drTRPM7-1258 HEK293 cells but without tetracycline induction (n = 5). The panel inset enlarges a portion of the left panel as indicated by the black box. (**C**) The respective IC_50_ values assessed in wild type and drTRPM7 truncation mutants and their respective S.D. are plotted according to length of the protein (Fig. 5A) (Hill_1478_ = 1.5; Hill_1258_ = 2; Hill_1178_ = 3). (**D**) Representative I/V curves of wild type and drTRPM7 truncation mutants extracted at 200 s of whole-cell recordings.

The first biophysical property we tested was the internal free Mg^2+^ sensitivity of the drTRPM7 mutants (Fig. 6). In whole-cell patch-clamp experiments, tetracycline-induced HEK293 T-REx cells were perfused with standard internal solution with increasing concentrations of intracellular Mg^2+^. Current amplitudes were extracted at −80 mV and +80 mV and 200 s into the experiment, normalized to cell size and plotted against the respective Mg^2+^ concentration for a subsequent dose-response curve fit (Fig. 6A). All drTRPM7 mutants developed TRPM7 currents over time with similar kinetics (Fig. 6B) and showed measurable currents with the signature current-voltage relationship of wild type drTRPM7 (Fig. 6D). drTRPM7-1178 developed enhanced currents at lower Mg^2+^ concentrations compared to wild type in analogy with its higher plasma membrane expression levels. Both drTRPM7-1478 and drTRPM7-1258 exhibited smaller overall currents at comparable internal Mg^2+^ concentrations (Fig. 6A,B,D). Since heteromerization of drTRPM7 with endogenous hTRPM7 cannot be excluded, and heterologous drTRPM7-1378 and 1258 mutants had smaller currents than wild type drTRPM7 in the presence of even small amounts of free intracellular Mg^2+^, we assessed endogenous hTRPM7-like currents at 844 μM intracellular free Mg^2+^ in drTRPM7-1258 control cells that had not been exposed to tetracycline (Fig. 6B). This showed that under these conditions endogenous hTRPM7 currents did not develop in any significant amount within the investigated time frame of 400 s, and in addition were about 9-fold smaller than drTRPM7-1548 currents in overexpressing cells. Interestingly, drTRPM7 mutants with either removal of the kinase domain or both the S/T-rich domain and the kinase domain showed increased sensitivity to internal Mg^2+^ compared to wild type as indicated by their respective IC_50_ (450 ± 0.55 μM and 250 ± 0.52 μM; Hill = 2 each; Fig. 6A,C). This effect was reversed to wild type values in the shortest drTRPM7 mutant that also had the CC region removed (840 ± 158 μM, Hill = 3; Fig. 6A,C).

### C-terminal truncation of drTRPM7 at amino acid 1178 removes inhibition by Mg·ATP

**Figure 7:**
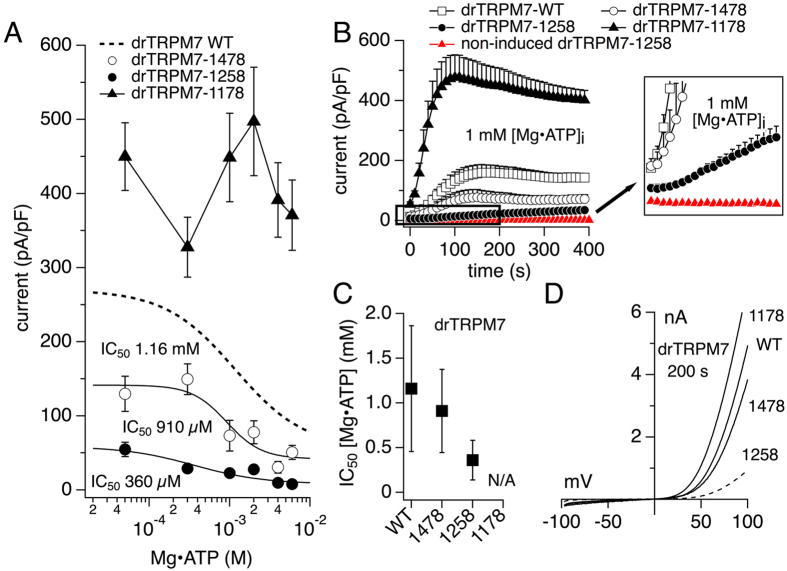
Truncation of drTRPM7 at amino acid 1178 eliminates inhibition by Mg·ATP. (**A**) drTRPM7 truncation mutants overexpressed in tetracycline-induced HEK293 T-REx cells were subjected to a Mg·ATP dose-response curve analysis using the whole-cell patch clamp technique with internal free Mg^2+^ clamped to 250 μM. drTRPM7 mutant currents were allowed to develop over time, amplitudes at +80 mV and 200 s were extracted, normalized for cell size, averaged and plotted versus the respective internal Mg·ATP concentration (n = 5 to 10). Error bars indicate s.e.m. Each respective dose-response curve was fitted to obtain their IC_50_. Dotted line represents the dose response fit for wild type drTRPM7 as shown in Fig. 1. (**B**) Whole-cell current development of tetracycline-induced heterologous wild type (replotted from Fig. 1D) and drTRPM7 truncation mutants in the presence of 250 μM internal Mg^2+^ and 1 mM Mg·ATP (in black; n = 5 to 10). Red symbols depict the current development of endogenous hTRPM7 under identical conditions in drTRPM7-1258 HEK293 cells but without tetracycline induction (n = 7). (**C**) The respective IC_50_ values assessed for Mg·ATP in wild type and drTRPM7 truncation mutants and their S.D. are plotted according to length of the protein (Hill_1478_ = 2; Hill_1258_ = 1). (**D**) Representative I/V curves of wild type and drTRPM7 truncation mutants with 1 mM internal Mg·ATP and extracted at 200 s of whole-cell recordings.

Next, we assessed the Mg·ATP-sensitivity of the tetracycline-induced drTRPM7 mutants overexpressed in HEK293 T-REx using the whole-cell patch-clamp technique. To this end, internal Mg^2+^ was held at 250 μM with increasing internal Mg·ATP levels in the standard internal solution (see methods). Current amplitudes were extracted at −80 mV and +80 mV and 200 s into the experiment, normalized to cell size and plotted against the respective Mg·ATP concentration. Similarly to the approach in Fig. 6B, endogenous hTRPM7 currents in HEK293 cells were assessed at 250 μM Mg^2+^ and 1 mM Mg·ATP (Fig. 7B) in non-induced drTRPM7-1258 cells. This revealed about 14-fold difference in current size between control and overexpressed drTPRM7-1258 cells at 200 s into the experiment, demonstrating negligible contaminating endogenous currents in overexpressing cells. At 1 mM intracellular Mg·ATP, drTRPM7-1478 developed currents with similar kinetics to wild-type, however, this concentration of Mg·ATP already fully inhibited drTRPM7-1258, and surprisingly did not seem to have any effect on the shortest mutant, drTRPM7-1178 (Fig. 7A,B). Further analysis revealed that only drTRPM7-1478 and drTRPM7-1258 exhibited a dose-response behavior to internal Mg·ATP, and corresponding curve fits confirmed their increasing sensitivity to Mg·ATP compared to wild type drTRPM7 (875 ± 457 μM and 221 ± 121 μM, respectively, compared to 1.16 ± 0.7 mM for wild type; Hill = 1; Fig. 7A,C). In addition, drTRPM7-1258 was about 4-fold more sensitive to intracellular Mg·ATP compared to the longer mutant drTRPM7-1478, as well as wild type (Fig. 7A,C). The shortest mutant drTRPM7 1178, on the other hand, had completely lost any sensitivity to intracellular Mg·ATP, and even 6 mM of the nucleotide was unable to affect drTRPM7-1178 currents in any significant way (Fig. 7A,C).

## Discussion

The experimental results presented here represent the first detailed characterization of the function and regulation of zebrafish TRPM7 (drTRPM7). The data demonstrate that Mg^2+^ and Mg·ATP inhibit drTRPM7 currents similarly to mammalian TRPM7 with calculated IC_50_ values of ~800 μM and 1.16 mM, respectively. Furthermore, drTRPM7 is similarly regulated by pH and high osmotic pressure. However, pharmacological profiling of drTRPM7 reveals that the underlying TRPM7 currents are facilitated by 2-APB rather than inhibited, and that the mammalian TRPM7 inhibitor waixenicin A has no effect on drTRPM7 (Table 1). Truncation mutants reveal that the channel domain upstream of aa 1178 retains a proper regulatory site for Mg^2+^ without further sensitivity to intracellular Mg·ATP. This indicates that the coiled-coil domain serves as a regulatory site for Mg-nucleotides.

The ubiquitously expressed TRPM7 has a central role in Mg^2+^ homeostasis and has been linked to many tissue-specific diseases^1^. Mammalian TRPM7 species investigated to date (human, mouse and rat) share similar biophysical characteristics. However, *in vivo* studies of TRPM7 are challenging, as the knockout of TRPM7 in mouse proves embryonically lethal^7^. In contrast, zebrafish survives a loss of function mutation in TRPM7, and instead causes defects in skeletogenesis and melanocyte death^22^. drTRPM7 shares 72% sequence identity with human TRPM7 and only 49% identity with human TRPM6 (www.uniprot.org).

Previous work with heterologous and/or native mouse and human TRPM7 reported constitutive regulation of these channels by intracellular Mg^2+^ and Mg·nucleotide triphosphates with IC_50_ in the range of ~750 μM and 2 mM, respectively^3^,^26^. While human TRPM6 (hTRPM6) is also highly sensitive to intracellular Mg^2+^, Mg·ATP is mandatory to sustain, rather than inhibit, channel function and prevent inactivation^14^. Thus, with its IC_50_ for Mg^2+^ and Mg·ATP of 800 μM and 1.2 mM, respectively, heterologous drTRPM7 compares well with regulation of mammalian TRPM7, both in regards to intracellular free Mg^2+^ and Mg·ATP (Fig. 1; Table 1). It should be noted that about 20% residual drTRPM7 currents remained active even at 6 mM intracellular Mg·ATP, thus partially mimicking the ATP-insensitive phenotype of hTRPM6 channels. This residual current is unlikely to be due to endogenous hTRPM6, as HEK293 cells do not express hTRPM6 natively^14^.

Human and mouse TRPM7, as well hTRPM6, are known to be divalent ion channels allowing the conductance of physiological and toxic metals into cells^5^,^12^,^27^. In mTRPM7, zinc (Zn^2+^) and nickel (Ni^2+^) are strongly preferred over Mg^2+^, and even Ca^2+^, while hTRPM6 prefers Zn^2+^ compared to all other divalent ions. Furthermore, while Mg^2+^ is conducted better than Ca^2+^ in hTRPM6, Ni^2+^ is the least preferred ion. The divalent profile assessment of drTRPM7 in the present study showed that cobalt (Co^2+^) was the preferred divalent, followed by Mg^2+^ and Ni^2+^. Similarly to mTRPM7, however, all divalent ions tested had a better permeation profile than Ca^2+^ itself (Fig. 2). Some of these differences might be explained on the amino acid level when looking at the channel’s pore region: Small amino acid changes before the EVY divalent cation filter^28^ may explain why mTRPM7, hTRPM6 and drTRPM7 have slightly different divalent profiles^4^,^5^,^29^. Overall, however, the results suggest that drTRPM7 conducts the same divalent ions as hTRPM7^27^ and mTRPM7 indicating that drTRPM7 represents a cellular entry point for these ions.

Mouse TRPM7 is modulated by both external pH and osmolarity^4^,^11^,^12^. The current study confirms these regulatory mechanisms also for drTRPM7 (Fig. 3). Specifically, drTRPM7-inward currents were strongly enhanced in an all-or-none fashion by acidic pH at and below 4.4. While this compares well with mTRPM7 and its EC_50_ of pH 4.5, mTRPM7 potentiation by pH proceeds in a graded fashion^4^. The pH effect in mouse is mediated by glutamate 1047, as a point mutation in that position leads to pH insensitivity. It is thought that acidic pH changes the selectivity filter of the mTRPM7 to preferentially allow monovalent ion influx, including protons^4^ and it is likely that this will also hold true for drTRPM7. In regards to osmotic changes, drTRPM7 currents were suppressed by hyperosmotic conditions with an IC_50_ of 655 ± 8.3 mOsm, which is aligned with mTRPM7 (IC_50_ = 460 mOsm^11^, but not hTRPM6, as the latter shows no sensitivity to osmotic changes^14^.

Two pharmacological agents have proven useful to differentiate between mammalian TRPM7 and TRPM6 currents: 2-Aminoethoxydiphenyl borate (2-APB) and waixenicin A. While the former inhibits both mTRPM7 and hTRPM7 in a non-selective way, it greatly enhances hTRPM6 currents^12^,^14^. On the other hand, waixenicin A represents a highly selective and potent inhibitor for human and rat TRPM7^13^, and has no effect on hTRPM6^14^. Surprisingly, the pharmacological profile of drTRPM7 mimics hTRPM6 rather than hTRPM7 in that waixenicin A had no effect on drTRPM7 while 2-APB greatly enhanced the currents at concentrations of 300 μM and above (Fig. 4; Table 1). Lower concentrations (30 μM and 100 μM) showed a small but statistically not significant inhibitory effect of similar magnitude, most likely due to native hTRPM7 channel activity endogenously expressed in HEK293 T-REx cells. Finally, the current-enhancing effect of 2-APB on heterologously expressed drTRPM7 could be confirmed with endogenous drTRPM7-like currents expressed in the native ZFL zebrafish liver cell line (Fig. 4). It should be noted that this current is unlikely to be carried by endogenous drTRPM6, as zebrafish liver tissue has poor expression of drTRPM6^23^. Thus, drTRPM7 shares excellent similarity to human TRPM6 in regards to pharmacology, which might indicate that the same protein region(s) may underlie both 2-APB and waixenicin A activity. This will make pharmacological intervention testing TRPM7’s involvement in a particular zebrafish disease model challenging. However, it also provides an opportunity to compare mammalian TRPM7, TRPM6 and drTRPM7 amino acid sequences, which may lead to the identification of regions involved in pharmacological regulation of the human TRPM7 protein.

Several studies have investigated the C-terminal coiled-coil (CC) regions of TRPM4, TRPM2 and TRPM8 and identified their involvement in protein expression, trafficking, subunit assembly and channel gating. Single or combined point mutations introduced into the *abcdef* heptad repeat in the CC domain of human TRPM2 (hTRPM2) revealed a direct correlation between tetrameric subunit assembly in the plasma membrane and size of ADPR-induced whole-cell currents^30^. Nevertheless, complete removal of the C-terminal CC region of hTRPM2 only suppressed and not abolished subunit assembly, since some adenosine diphosphate ribose (ADPR)-induced activation of hTRPM2 currents was retained^31^. A C-terminal truncation of 160 amino acids in human TRPM4 (hTRPM4) that included the CC region did not prevent hTRPM4 localization to the plasma membrane^32^. However, both heteromerization with wild type hTRPM4 and channel gating by intracellular Ca^2+^ at physiological voltages was absent. This indicated that the CC region of TRPM4 might be required for proper channel assembly. Interestingly, deletions or truncations within the C-terminal region of hTRPM4 that included the CC region strongly influenced the channel’s gating behavior by reducing intracellular Ca^2+^ sensitivity and shifting the activation potential beyond +100 mV^33^. In human TRPM8 (hTRPM8) a single point mutation in the channel’s CC region (L1089P) did not affect channel trafficking to the plasma membrane but was sufficient to strongly reduce oligomerization of hTRPM8 monomers^34^. Work using rat TRPM8 (rTRPM8) as model showed that the C-terminal CC region not only supported assembly of functional channels in the plasma membrane but also was required for channel activity regulation in response to ligands or cold^35^,^36^. On the other hand, the crystal structure of the rat TRPM7 (rTRPM7) CC domain revealed a four-stranded antiparallel coiled-coil structure with *a-d* core packing^37^. Further analysis of these data allowed structural bundling of rTRPM7 CC domains into two distinct groups that also followed phylogenetic origin: the TRPM7 group (TRPM1, TRPM3, TRPM6 and TRPM7) and the TRPM8 group (TRPM2, TRPM4, TRPM5 and TRPM8). It was further noted that the CC domain of rTRPM6 and rTRPM7 were the most similar to each other, whereas the CC of rTRPM8 not only is shorter, but also rather poorly conserved in comparison to rTRPM7’s antiparallel CC domain arrangement^37^.

Our data show that in zebrafish TRPM7 (drTRPM7) removal of the C-terminus that includes the coiled-coil domain was able to rescue TRPM7-deficient cell growth inhibition (Fig. 5) and rendered a functional channel, albeit with altered gating properties. While the channel retained regulation through intracellular Mg^2+^ similar to wild type (Fig. 6), it lost regulation by Mg·ATP (Fig. 7). This indicates that drTRPM7 subunits lacking the CC domain are expressed, are trafficked to, and form functional channel oligomers in the plasma membrane. Thus it seems that the CC domain in drTRPM7 is primarily concerned with channel regulation rather than channel subunit assembly. Similar to Shaker potassium channels, the channel’s transmembrane domain may prove sufficient for channel subunit assembly in the plasma membrane^38^.

It is likely that endogenous hTRPM7 expressed by HEK293 cells and heterologous drTRPM7 will form heteromeric channels. The dose-response behavior of truncation mutants might be affected by such heteromerization (Figs 6 and 7). Nevertheless, such interaction would be limited by the amount of available endogenous hTRPM7 subunits^14^. We therefore repeated the experimental conditions shown in Fig. 6B (844 μM free internal Mg^2+^) and Fig. 7B (1 mM internal Mg·ATP) in HEK293 T-REx cells stably expressing the drTRPM7-1258 mutant, but without tetracycline induction to assess endogenous hTRPM7 current development. This revealed that for both conditions cells did not develop endogenous TRPM7-like currents over the time observed, and that tetracycline-induced currents exceeded endogenous currents by at least 10-fold.

Previous work on the role of drTRPM7 in zebrafish escape behavior identified three alleles in the zebrafish mutant *touchdown* harboring mutations that yield non-functional channels when expressed in *Xenopus* oocytes and assessed with two- electrode voltage-clamp (TEV)^20^. While one mutant has a point mutation in the second transmembrane domain of drTRPM7, another truncates the protein shortly thereafter at aa R891. However, the third *touchdown* mutant allele introduces a drTRPM7 kinase domain deletion, truncating the protein at aa 1410. In contrast, our three truncation mutants show functional activity both in regards to heterologous drTRPM7 currents (Figs 6 and 7) as well as rescue of growth arrest in chicken DT40 B cells with a TRPM7 knock-out^6^ (Fig. 5). A critical difference between *Xenopus* oocyte electrophysiology and whole-cell patch clamp is the ability to control the intracellular milieu during data acquisition. Our whole-cell experiments identify an increased sensitivity of the drTRPM7-1478 and drTRPM7-1258 truncation mutants to intracellular Mg^2+^ (Fig. 6). It is therefore conceivable that the drTRPM7-1410 kinase domain deletion *touchdown* mutant also has a heightened sensitivity to intracellular Mg^2+^, hence the inability to assess its current-voltage behavior in *Xenopus* oocytes.

In human TRPM7, mutating K1648 or G1799 eliminates the phosphorylation activity of the kinase domain and renders an ion channel with reduced sensitivity to intracellular Mg^2+^ and relative insensitivity to intracellular Mg-nucleotides (Mg·NTP)^6^. Surprisingly, however, truncating the kinase domain at aa 1569 again increases Mg^2+^- and Mg·ATP-sensitivity compared to wild-type, while removing discrimination between Mg-NTP species^26^. It was therefore proposed that Mg·ATP-dependent regulation of TRPM7 is mediated by the catalytic site on the kinase domain, which synergistically interacts with a Mg^2+^ regulatory site further upstream, the latter also allowing indiscriminate Mg·NTP sensitivity. Evidence for two Mg^2+^ regulatory sites also emerges from whole-cell experiments in native cell systems (human Jurkat T lymphocytes and HEK293 cells^39^), as well as elegant single-channel analyses in cell-free patches in Jurkat T cells^40^. In mouse, a downstream TRPM7 kinase truncation at aa 1599 yields a non-functional channel^24^, while cutting mTRPM7 at aa 1510 regains regular-sized currents with a current-voltage behavior typical for TRPM7^25^. This argues for the importance of this region for regulation by Mg^2+^. Furthermore, mutating S1107 in mTRPM7’s TRP domain produces a constitutively active ion channel that cannot be inhibited by internal Mg^2+ ^41. Whether the S1107E mutant retains sensitivity to Mg·NTPs remains unknown.

Analysis of our three drTRPM7 truncation mutants reveals that the postulated high-affinity Mg^2+^ regulatory site^6^ may reside within the coiled-coil region in zebrafish, since the drTRPM7-1258 retains the highest Mg^2+^ sensitivity compared to the other mutants. In addition, removal of the coiled-coil region exposes a third Mg^2+^-sensitive site upstream of the aa 1178 truncation, as the channel retains sensitivity to intracellular Mg^2+^ inhibition that is comparable to wild type drTRPM7. In zebrafish, this likely may involve aa S1088, the equivalent position to S1107 in mouse^41^, and this position seems conserved in mouse, human and zebrafish. Importantly, the Mg^2+^-sensitive site within the coiled-coil region also retains regulation by Mg·ATP, which is strongest in the drTRPM7-1258 mutant (Fig. 7). Removal of the C-terminus including the coiled-coil region, however, completely eliminates Mg·ATP-mediated inhibition of the channel. This indicates that the third Mg^2+^-regulated site upstream of the coiled-coil domain is a pure Mg^2+^ sensor. Thus, we can expand our hypothetical model of Mg^2+^ and Mg·nucleotide regulation of TRPM7^26^ such that the wild type TRPM7 has three distinct intracellular Mg^2+^ sensitive sites: within the kinase domain^6^, within the coiled-coil region (Figs 6 and 7), and a third regulatory site possibly involving S1088^41^.

## Additional Information

**How to cite this article**: Jansen, C. *et al*. The coiled-coil domain of zebrafish TRPM7 regulates Mg·nucleotide sensitivity. *Sci. Rep.* **6**, 33459; doi: 10.1038/srep33459 (2016).

## Figures and Tables

**Table 1:** Biophysical characteristics of heterologous mammalian TRPM7, hTRPM6 and drTRPM7.

Characteristic	Mammalian TRPM7	Human TRPM6	drTRPM7
Mg^2+^	IC_50_ 720 μM^26^	IC_50_ 29 μM^14^	IC_50_ 778 μM
Mg·ATP	IC_50_ 2 mM^26^	No effect^14^	IC_50_ 1.16 mM
Divalent Profile	Non-specific divalent permeability^5^	Non-specific divalent permeability^4^	Non-specific divalent permeability
pH	Acidic pH potentiates^4^	Acidic pH potentiates^4^	Acidic pH potentiates
Osmolarity	Inhibition^11^	No effect^14^	Inhibition
2-APB	Inhibition^12^	Potentiation^12^	Potentiation
Waixenicin A	Inhibition^13^	No effect^14^	No effect
